# Disruption of focal adhesion kinase and p53 interaction with small molecule compound R2 reactivated p53 and blocked tumor growth

**DOI:** 10.1186/1471-2407-13-342

**Published:** 2013-07-11

**Authors:** Vita M Golubovskaya, Baotran Ho, Min Zheng, Andrew Magis, David Ostrov, Carl Morrison, William G Cance

**Affiliations:** 1 Department of Surgical Oncology, Roswell Park Cancer Institute, Buffalo, NY 14263, USA; 2 The University of Oklahoma Cell Sciences Center, Gainesville, FL, USA; 3 Shands Cancer Center, University of Florida, Gainesville, FL, USA; 4 Department of Pathology, Roswell Park Cancer Institute, Buffalo, NY 14263, USA

**Keywords:** Focal adhesion kinase, p53Cancer, Small molecule, p21, Tumor, Apoptosis

## Abstract

**Background:**

Focal Adhesion Kinase (FAK) is a 125 kDa non-receptor kinase that plays a major role in cancer cell survival and metastasis.

**Methods:**

We performed computer modeling of the p53 peptide containing the site of interaction with FAK, predicted the peptide structure and docked it into the three-dimensional structure of the N-terminal domain of FAK involved in the complex with p53. We screened small molecule compounds that targeted the site of the FAK-p53 interaction and identified compounds (called Roslins, or R compounds) docked *in silico* to this site.

**Results:**

By different assays in isogenic HCT116p53^+^/^+^ and HCT116 p53^-^/^-^ cells we identified a small molecule compound called Roslin 2 (R2) that bound FAK, disrupted the binding of FAK and p53 and decreased cancer cell viability and clonogenicity in a p53-dependent manner. In addition, dual-luciferase assays demonstrated that the R2 compound increased p53 transcriptional activity that was inhibited by FAK using p21, Mdm-2, and Bax-promoter targets. R2 also caused increased expression of p53 targets: p21, Mdm-2 and Bax proteins. Furthermore, R2 significantly decreased tumor growth, disrupted the complex of FAK and p53, and up-regulated p21 in HCT116 p53^+^/^+^ but not in HCT116 p53^-^/^-^ xenografts *in vivo.* In addition, R2 sensitized HCT116p53^+^/^+^ cells to doxorubicin and 5-fluorouracil.

**Conclusions:**

Thus, disruption of the FAK and p53 interaction with a novel small molecule reactivated p53 in cancer cells *in vitro* and *in vivo* and can be effectively used for development of FAK-p53 targeted cancer therapy approaches.

## Background

Focal Adhesion Kinase (FAK) is a non-receptor tyrosine kinase that controls cellular processes such as proliferation, adhesion, spreading, motility, and survival [1-6]. FAK is over-expressed in many types of tumors [7-10]. We have shown that FAK up-regulation occurs in the early stages of tumorigenesis [11]*.* Real-time PCR analysis of colorectal carcinoma and liver metastases demonstrated increased FAK mRNA and protein levels in tumor and metastatic tissues versus normal tissues [10]*.* Cloning and characterization of the FAK promoter demonstrated different transcription factor binding sites, including p53 that repressed FAK transcription [12,13]*.* In addition, analysis of 600 breast cancer tumors demonstrated a high positive correlation between FAK overexpression and p53 mutations [14,15]*.* Recently, p53-dependent repression of FAK has been demonstrated in response to estradiol in breast cancer cells [16]*.* Thus, FAK and p53 signaling pathways are cross-linked in cancer [12,17]*.*

Recently we have demonstrated a direct interaction of the p53 protein with the N-terminal domain of FAK [18]. We performed mapping analysis and have shown that the N-terminal domain of FAK binds the N-terminal domain of p53 (from 1 to 92 a.a) [18]. The binding of FAK and p53 has been demonstrated in different cancer cell lines: cells as well as normal human fibroblasts [18]*.* In addition, we have shown that overexpressed FAK inhibited p53-induced apoptosis in SAOS-2 cells and decreased p53-mediated activation of p21, BAX, and MDM-2 targets in HCT116 p53^+^/^+^ cells [18] The interaction of FAK and p53 has been confirmed by another group, who demonstrated that FAK interacted with p53 to down-regulate its signaling [19]. These observations are consistent with FAK’s role in sequestering proapoptotic proteins to enhance survival signaling [15]. We next identified the 7 amino-acid binding site in the proline-rich region of p53 protein (amino-acids 65–72) that is involved in interaction with FAK [20]. In addition, the p53 peptide containing this binding site was able to disrupt the binding of FAK and p53, to activate p53 and to inhibit viability of HCT116p53^+^/^+^ cells compared to HCT116p53^-^/^-^ cells, suggesting that FAK-p53 targeting can be used for therapeutics [20]. A recent review provided a model of the FAK and p53 interaction, where the FERM N-terminal domain of FAK mediated signaling between the cell membrane and the nucleus [21].

Reactivation of p53 is critical for development of p53-targeted therapeutics [22]. It is estimated that approximately 50% of human cancers express wild type p53, and p53 is inactivated in these tumors by different mechanisms [22,23]. There were several reports on reactivation of p53 with different compounds that disrupted the Mdm-2 and p53 complex [24-29]. In fact, most studies that report reactivation of p53 have focused only on the p53-MDM-2 interaction. However, FAK binds to both p53 and MDM-2 and is a key component of this complex [15]. As FAK sequesters p53, it inactivates p53 repression of its promoter, resulting in more FAK in the tumor cell [15]. Thus, one of the novel mechanisms inactivating p53 function is overexpression of FAK in tumors [18,30]. These observations from the rationale for disrupting this interaction and reactivating p53 tumor suppressor functions.

In this report, we sought to identify small molecule drug-like compounds that disrupted FAK and p53 binding and caused p53-dependent cytotoxicity and tumor cells. We performed a three-dimensional computer modeling of the p53 peptide structure involved in interaction with FAK [20] and docked this p53 peptide into the three-dimensional crystal structure of FAK-NT, reported in [31]. We generated a model of the FAK and p53 interaction and performed screening of >200,000 small molecule compounds from the National Cancer Institute database, which were docked into the region of the FAK and p53 interaction. We called these compounds Roslins (from Roswell Park Cancer Institute) and identified a lead small molecule compound R2: 1-benzyl-15,3,5,7-tetraazatricyclo[3.3.1.1~3,7~] decane, that bound to the FAK-N-terminal domain and disrupted the FAK and p53 complex. The R2 compound decreased viability and clonogenicity of HCT116 cells in a p53-dependent manner, and reactivated FAK-inhibited transcriptional activity of p53 with p21, Mdm-2 and Bax transcriptional targets. The combination of R2 and either doxorubicin, or 5-fluorouracil further decreased cancer cell viability more efficiently than each inhibitor alone in HCT116 cells in a p53-dependent manner and reactivated p53-targets. Thus, targeting the FAK and p53 interaction with small molecule inhibitor R2 can be a novel therapeutic approach to reactivate p53 and decrease cancer cell viability, clonogenicity and tumor growth.

## Methods

### Cell lines and culture

The HCT116p53^-^/^-^ and HCT116p53^+^/^+^ colon cancer cells were obtained from Dr. Bert Vogelstein (Johns Hopkins University) and maintained in McCoy’s5A medium with 10% FBS and 1 μg/ml penicillin/streptomycin. The HCT116 cell lines were authenticated by Western blotting with p53 antibody and passaged less than 6 month after resuscitation of frozen aliquots. MCF-7, PANC-1, and SW620 cells were obtained from ATCC and cultured according to the manufacturer’s protocol. The cell lines were passaged less than 6 month after resuscitation of frozen aliquots.

### Antibodies

The FAK monoclonal FAK (4.47) antibody was purchased from *Upstate Biotechnology,* phospho-Y397-FAK antibody was obtained from *Biosource Inc.* Monoclonal anti-*β-*actin antibody was obtained from *Sigma.* Anti-p53 antibody (Ab-6, clone DO-1) was obtained from *Oncogene Research Inc.* p21, Mdm-2 and Bax antibodies were obtained from *Santa Cruz.*

### Plasmids and reagents

The p21-pGL3, BAX-pGL3 and Mdm-2-pGL2 promoter luciferase constructs, were described previously [18]. The recombinant baculoviral FAK [18] was used for pull-down assay. The FAK-NT (1–422 aa) fragment was subcloned into the pET200 vector (*Invitrogen*) and the His-tagged FAK-NT protein was isolated according to the instructions of the Ni-NTA Purification system kit (*Invitrogen*). The recombinant p53 was obtained from *BD Pharmingen.* The R2 compound (1-benzyl-15,3,5,7-tetraazatricyclo [3.3.1.1~3,7~] decane) was kindly provided by Drs. Ethirajan Manivannan and Ravindra Pandey. A18 compound (1,4-bis(diethylamino)-5,8-dihydroxy anthraquinon) [32] and M13 compound (5′-O-Tritylthymidine) [33] were obtained from NCI and Sigma, respectively.

### Peptide docking

We used a structure-based approach combining docking of FAK and p53 peptide interaction and molecular docking of small molecule compounds with functional testing, as described [33]. Initially, we predicted the three dimensional structure of the p53 region involved in interaction with FAK in the N-terminal domain of p53 by the PHYRE (Protein Homology/analog Y Recognition Engine) server (http://www.sbg.bio.ic.ac.uk/phyre) [34]. PHYRE is an efficient protein structure prediction method by sequence homology to existing structures [34]. While the portion of the p53 region described [35] was successfully modeled by the PHYRE server, the region, which involved in interaction with FAK-NT [20] was predicted as disordered. We therefore isolated the disordered seven-amino-acid peptide (RMPEAAP) known to be involved in interaction with FAK [20] from the model, assigned residue charges and add hydrogen atoms with the UCSF CHIMERA program and performed flexible docking to the FAK-FERM domain by DOCK 6.0 software to find the highest scoring complex of FAK and p53 peptide. The crystal structure of FAK, N-terminal FERM domain (PDB ID:2AL6), reported [31] was used for docking and computer modeling of the FAK and p53 peptide interaction. To model the FAK-NT-p53 peptide interaction, the DOCK 6.0 software analyzed >10,000 possible orientations of this interaction, based on the scores of the resulting interfaces using electrostatics (ES) and van der Waals (vWS) energies. The model with the highest scoring of FAK-NT and p53 peptide interaction has been generated and compared with the FAK lobes amino acids reported recently to interact with FAK [19], and FAK-NT region [20]. All binding poses were evaluated using the DOCK grid-based scoring, involving the non-bonded terms of the AMBER molecular mechanics force field (vDW+ES).

### Molecular docking of small molecule compounds

More than 200,000 small-molecule compounds from National Cancer Institute Development Therapeutics Program NCIDTP library (http://dtp.nci.nih.gov) [36] and compounds from ZINC UCSF (University of California, San Franscisco) database (http://zinc.docking.org/catalogs/ncip (version 12) [37] following the Lipinski rules were docked into the pocket of the N-terminal domain of FAK and p53 interaction in 100 different orientations using the DOCK5.1 program. The spheres describing the target pocket of FAK-p53 were created using the DOCK 5.1 suite program SPHGEN. Docking calculations were performed on the University of Florida High Performance Computing supercomputing cluster (http://hpc.ufl.edu). Scores were based on a grid spaced five angstroms from the spheres selected for molecular docking. Each compound was docked in 100 orientations, and grid-based energy scores were generated for the highest scoring orientations. Scores approximate delta G values based on the sum of polar electrostatic interactions and van der Waals energies. Small molecule partial atomic charges were calculated using the SYBDB program, as described [38,39].

### Small molecule compounds

The top compounds that were detected by the DOCK5.1 program to best fit into FAK-p53 pocket were ordered from the NCI/DTP database free of charge. Each of the compounds (called Roslin compounds) was solubilized in water or DMSO at a concentration of 25 mM. The R2 compound was chemically synthesized for biochemical analyses *in vitro* and for mice studies *in vivo*.

### Clonogenicity assay

The 1000 cells were plated on 6 well plates and incubated with or without tested compound for 1–2 weeks. Then cells were fixed in 25% methanol and stained with Crystal Violet, and colonies were visualized and counted.

### Cell viability assay

The cells (1×10 4 cells per well) were plated on a 96 well plate and after 24 hours treated with compounds at different concentrations for 24 hours. The 3-(4,5-dimethylthiazol-2-yl)-5-(3-carboxymethoxyphenyl)-2-(4-sulfophenyl)-2H-tetrazolium compound from Promega Viability kit (Madison, IL) was added, and the cells were incubated at 37C for 1–2 hours. The optical density at 490 nm on 96-plate was analyzed with a microplate reader to determine cell viability.

### Western blotting, immunoprecipitation and immunostaining

Western blotting, immunoprecipitation, immunostaining and immunohistochemical staining using were performed, as described [40].

### Pull-down assay

For the pull-down assay we used recombinant baculoviral FAK, GST and GST-p53 proteins, as described [18] and performed pull-down assay, as described [20].

### Octet RED binding

The binding was performed by ForteBio Inc. company (http://www.fortebio.com). The human FAK-N-terminal domain protein was biotinylated using NHS-PEO4-biotin (Pierce). Superstreptavidin (SSA) biosensors (*FortéBio Inc.*, Menlo Park, CA) were coated in a solution containing 1 μM of biotinylated protein. A duplicate set of sensors was incubated in an assay buffer (1× kinetics buffer of *ForteBio Inc.*) with 5% DMSO without protein for use as a background binding control. Both sets of sensors were blocked with a solution of 10 mg/ml Biocytin for 5 minutes at 25°C. A negative control of 5% DMSO was used. The binding of samples (500 μM) to coated and uncoated reference sensors was measured over 120 seconds. Data analysis on the *FortéBio Octet RED* instrument was performed using a double reference subtraction (sample and sensor references) in the *FortéBio* data analysis software.

For detection of FAK and p53 protein dissociation by R2 compound, p53 protein was biotinylated and bound to the streptavidin biosensor at 25 μg/ml. Then 500 nM FAK-NT was used for association and dissociation step in a 1× kinetics buffer, either without R2 or with R2 at 111, 333 or 1000 μM. The association and dissociation plot and kinetic constants were obtained with *FortéBio* data analysis software.

### Dual luciferase assay

The dual-luciferase was performed, as described (18). In brief, 2×10^5^cells were plated on 6-well plates, and co-transfected with the p21, Mdm-2 or Bax promoters in the pGL2 or pGL3-luciferase containing plasmids (1 μg/well) and pPRL-TK plasmid containing the herpes simplex virus thymidine kinase promoter encoding Renilla luciferase (0.1 μg/well) using Lipofectamine (*Invitrogen*) transfection agent according to the manufacture’s protocol. HCT116 p53^-^/^-^ cells were co-transfected with the above plasmids and p53 in the presence or absence of FAK plasmids and tested either without or with 25 microM R2 compound for 24 h.

### FACS analysis

Flow cytometry analysis was performed by the standard protocol at Roswell Park Flow Cytometry Core Facility. The percentage of G1, G2, S phase-arrested and/or apoptotic cells was calculated.

### Tumor growth in nude mice *in vivo*

Female nude mice, 6 weeks old, were obtained from Harlan Laboratory. The mice experiments were performed in compliance with IACUC protocol approved by the Roswell Park Cancer Institute Animal Care Committee. HCT116 p53^+^/^+^ and p53 ^-^/^-^ cells (3.7×10^6^ cells/injection) were injected subcutaneously into the right and left leg side of the same mice, respectively. Three days after injection, the R2 compound was introduced by IP injection at 60 mg/kg dose daily 5 days/week. Tumor diameters were measured with calipers and tumor volume was calculated using this formula = (width)^2^×Length/2).

### Statistical analyses

Student’s t test was performed to determine significance. The difference between treated and untreated samples with P<0.05 was considered significant.

## Results

### Computer modeling revealed compounds targeting the FAK-p53 interaction

**Figure 1:**
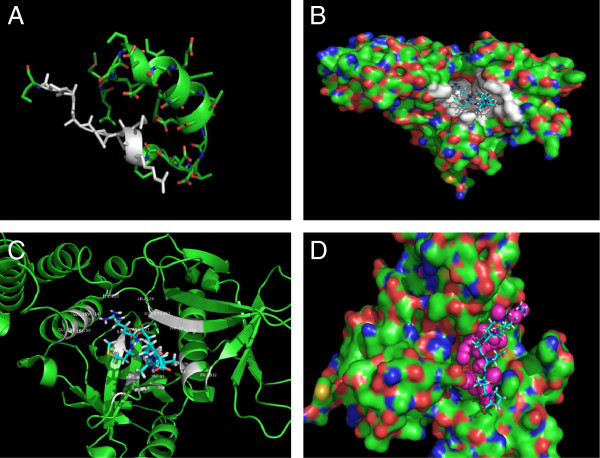
**The computer modeling and docking of p53 peptide involved in interaction with FAK and small molecules targeting FAK-p53 interaction. A.** The secondary structure of p53 peptide (43–73 aa) predicted with PHYRE (Protein Homology/analogy recognition engine), as described [34]. The 7 amino-acid p53 peptide (65–72 amino acids of p53) found to be involved in interaction with FAK [20] is shown by grey color. **B.** The docking of the 7 amino acid p53 peptide involved in interaction with FAK inside the crystal structure of FAK-NT (N-terminal domain of FAK). The amino acids of FAK-NT interacting with the 7 amino acid p53 peptide are shown in white color. **C.** Zoomed image of FAK-NT interaction with the 7 amino acid p53 peptide. The amino-acids of FAK interacting with p53 peptide: R86, V95, W97, R125, I126, R127, L129, F147, Q150, D154, E256, F258, K259, P332, I336 and N339. **D.** Small molecules targeting FAK-p53 interaction. Screening of NCI small molecule database with DOCK5.1 program identified small molecules (called R compounds) docked into the region of FAK and p53 interaction. The purple color marks small molecule spheres. Peptide is shown by blue color and FAK-NT by green color.

We detected the 7 amino-acid region in p53 involved in the interaction with FAK [20], and because the crystal structure of this N-terminal region of p53 remained unsolved, we performed computer modeling with a PHYRE program (Protein Homology/analog Y Recognition Engine) that allowed us to predict its three-dimensional structure, based on protein homology and an analogy recognition engine [18]. The region containing 43 to73 amino acids of the N-terminal proline-rich domain of p53 had an alpha-helical conformation and contained the 7 amino-acid peptide involved in the interaction with FAK) (Figure 1A). We performed docking of the 7 amino acid p53 peptide (65–71 amino acids) involved in interaction with FAK into the N-terminal domain of FAK and found the best complex of FAK and p53 peptide (Figure 1B). The model with the highest scoring of the FAK N-terminal domain (FAK-NT) and p53 peptide interaction was created, which included amino-acids from the F1 (33–127 aa) and F2 lobes (128–253 aa) of FAK reported to interact with p53 [19] (Figure 1C).

To find small molecule compounds targeting the FAK and p53 interaction we screened more than 200,000 small-molecule compounds from the National Cancer Institute database and docked them into the region of FAK-p53 interaction (Figure 1D). We identified a series of small molecule compounds that we called Roslins that effectively docked into the FAK-p53 interaction region (Figure 1D). The p53 peptide (blue color) and small molecules (purple color) which target the region of FAK and p53 interaction are shown in Figure 1D.

### The small molecule compound R2 decreased HCT116 viability and clonogenicity in a p53- and dose-dependent manner

**Figure 2:**
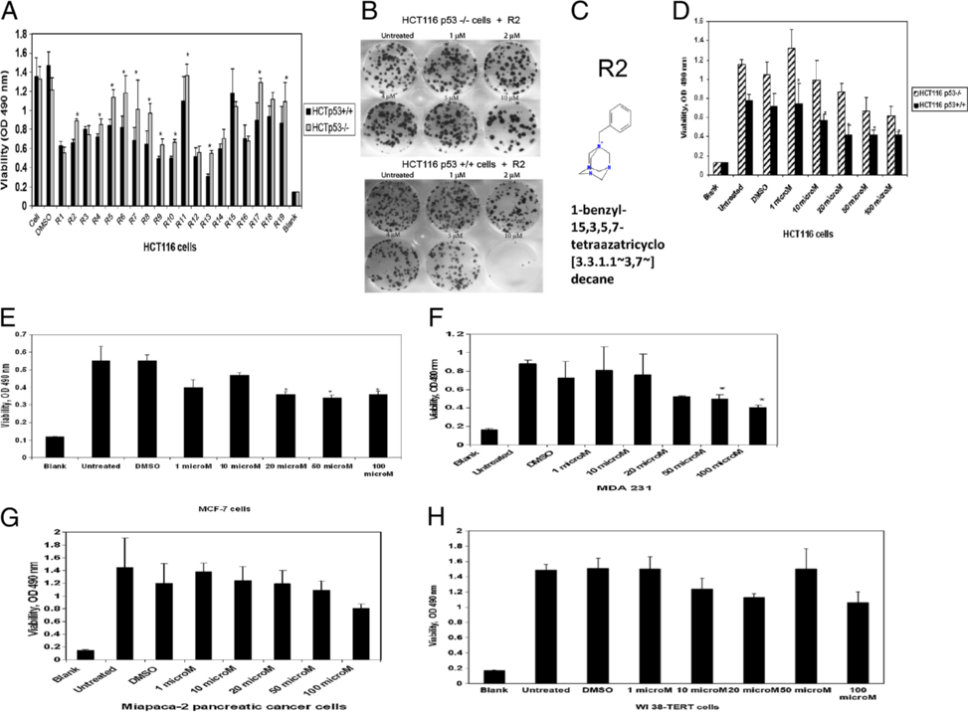
**R2 is a lead compound targeting FAK-p53 interaction. A.** The viability MTT assay with HCTp53^+^/^+^ and HCTp53^-^/^-^ cells identified small molecules, called R compounds that significantly decreased the viability of HCT116 p53^+^/^+^ cells compared with HCT116p53^-^/^-^ cells. * P<0.05 viability less in HCT116p53^+^/^+^ cells versus HCT116 p53^-^/^-^ cells. **B.** R2 significantly decreased cancer cell clonogenicity in a p53-dependent manner. The compound R2 decreased clonogenicity in HCT116p53^+^/^+^ cells more significantly than in HCT116p53^-^/^-^ cells. **C.** The structure of R2 compound. **D.** The R2 compound decreased cancer cell viability in a p53-and dose-dependent manner. MTT assay with different doses of R2 compound was performed in HCT116p53^+^/^+^ and HCT116p53^-^/^-^ cells. *p<0.05, R2-treated HCT116p53^+^/^+^ versus HCT116 p53^-^/^-^ cells. **E, F.** R2 compound decreased the viability of cancer cell lines with wild type p53 more efficiently than with mutant p53. MTT assay was performed with different doses of R2 in MCF-7 (wild type p53) **(E)** and MDA231 (mutant p53) **(F)** breast cancer cells. * p<0.05 treated with R2 versus untreated cells. **G**. MTT assay with R2 in pancreatic cancer cell line, Miapaca-2 cells (mutant p53). **H.** MTT assay with R2 in normal human WI 38-hTERT fibroblasts. The MTT assay was performed as in Figure 2 E, F.

We selected 19 compounds targeting the FAK and p53 interaction, R1 to R19 (Table 1), and tested them for p53-dependent decrease of cell viability in HCT116p53^+^/^+^ and HCT116p53^-^/^-^ cells (Figure 2A). The R2, R4-R11, R13, R17 and R18 compounds decreased HCT116 p53^+^/^+^ cell viability more efficiently than in HCT116 p53−/− cells (Figure 2A). Most of these compounds also decreased viability in a A375 melanoma cancer cell line with wild type p53 (Additional file 1: Figure S1). Then we tested R compounds that decreased viability in a p53-dependent manner for disruption of FAK and p53 interaction by immunoprecipitation of FAK and p53. Among these compounds R2, R5-R10 and R13 effectively disrupted FAK and p53 interaction. To test specificity for the FAK and p53 pathway we used control p53-null MEF FAK^-^/^-^ cells and PANC-1 with mutant p53, as negative controls (Additional file 1: Figure S1). As expected, most of these compounds did not affect viability of control FAK^-^/^-^p53^-^/^-^ cells, except for R9, R10 and R13 or PANC-1 cells with mutant p53, except for R9,R10 and R13 (Additional file 1: Figure S1). Thus, among all R compounds, R2, R5, R6, R7, and R8 were the most specific compounds in targeting the FAK and p53 interaction and pathway.

**Table 1:** Top scoring FAK-p53 targeting compounds: R compounds

**Comp No**	**Comp Label**	**NSC**	**Formula**	**Molecular Weight**	**Name**
1	R1	5754	C14H14O3	230	2-(3-methylbutanoyl)-1H-indene-1,3(2H)-dione
2	**R2**	**10408**	**C13H19N4**	**231**	**1-benzyl-15,3,5,7-tetraazatricyclo[3.3.1.1~3,7~]decane**
3	R3	32237	C14H18BrN4O	338	1-(4-bromophenyl)-2-(15,3,5,7-tetraazatricyclo[3.3.1.1~3,7~]dec-1-yl)ethanone
4	R4	32895	C14H18ClN4O	294	1-(4-chlorophenyl)-2-(15,3,5,7-tetraazatricyclo[3.3.1.1~3,7~]dec-1-yl)ethanone
5	R5	33450	C14H17Cl2N4O	328	1-(2,4-dichlorophenyl)-2-(15,3,5,7-tetraazatricyclo[3.3.1.1~3,7~]dec-1-yl)ethanone
6	R6	34564	C14H18IN4O	385	1-(4-iodophenyl)-2-(15,3,5,7-tetraazatricyclo[3.3.1.1~3,7~]dec-1-yl)ethanone
7	R7	34740	C15H21N4O2	289	1-(4-methoxyphenyl)-2-(15,3,5,7-tetraazatricyclo[3.3.1.1~3,7~]dec-1-yl)ethanone
8	R8	35024	C14H19IN5O	400	1-(4-iodophenyl)-2-(15,3,5,7-tetraazatricyclo [3.3.1.1~3,7~]dec-1-yl)ethanone oxime
9	R9	35450	C20H23N4O	335	1-[1,1′-biphenyl]-4-yl-2-(15,3,5,7-tetraazatricyclo[3.3.1.1~3,7~]dec-1-yl)ethanone
10	R10	36400	C18H21N4O	309	1-(2-naphthyl)-2-(15,3,5,7-tetraazatricyclo[3.3.1.1~3,7~]dec-1-yl)ethanone
11	R11	36791	C18H25N4O	313	2-(15,3,5,7-tetraazatricyclo[3.3.1.1~3,7~]dec-1-yl)-1-(5,6,7,8-tetrahydro-2-naphthalenyl)ethanone
12	R12	80640	C13H18BrN4	310	1-(4-bromobenzyl)-15,3,5,7-tetraazatricyclo[3.3.1.1~3,7~]decane
13	R13	141562	C17H22N5	296	1-((2-methyl-3-quinolinyl)methyl)-15,3,5,7-tetraazatricyclo[3.3.1.1~3,7~]decane
14	R14	155877	C13H16Cl3N4	334	1-(2,4,5-trichlorobenzyl)-15,3,5,7-tetraazatricyclo[3.3.1.1~3,7~]decane
15	R15	168615	C9H15Br2N4	339	1-(2,3-dibromo-2-propenyl)-15,3,5,7-tetraazatricyclo[3.3.1.1~3,7~]decane
16	R16	254980	C10H24N4	200	3,6-dibutyl-1,2,4,5-tetraazinane
17	R17	281702	C12H24N4	224	N-butyl-N-methyl-1,3,5-triazatricyclo[3.3.1.1~3,7~]decan-7-amine
18	R18	281707	C14H28N4	252	N-hexyl-N-methyl-1,3,5-triazatricyclo[3.3.1.1~3,7~]decan-7-amine
19	R19	407323	C18H21N4O	309	1-(1-naphthyl)-2-(15,3,5,7-tetraazatricyclo[3.3.1.1~3,7~]dec-1-yl)ethanone

R2 compound marked in bold font.

To test these compounds for long-term effects, we performed clonogenicity assays in HCT116 p53^+^/^+^ and HCT116p53^-^/^-^ cells. Among the R compounds targeting FAK and p53, R2 compound (Table 1, marked in bold) maximally decreased clonogenicity in HCT116p53^+^/^+^ (Additional file 2: Figure S2). The R2 compound decreased clonogenicity in a p53- and dose-dependent manner (Figure 2B). The structure of R2 is shown on Figure 2C. R2 also decreased viability of HCT116 cell in a p53- and dose-dependent manner (Figure 2D). Thus, the small molecule compound R2 was selected for further study because it decreased viability and clonogenicity in a dose and p53-dependent manner in HCT116 cells.

### R2 compound decreased viability in cancer cells with wild type p53 more effectively than in cancer cells with mutant p53 or in normal cells

We tested the effect of the R2 compound on viability of the MCF-7 breast cancer cell line with wild type p53. R2 decreased viability in the MCF-7 cells in a dose-dependent manner (Figure 2E). In the MDA-231 breast cancer cell line with mutant p53, R2 also decreased viability, but the significantly decreased viability was observed at higher dose than in cells with wild type p53: 50 μM in MDA-231 (Figure 2F) versus 20 μM in MCF-7 cells (Figure 2E). R2 did not significantly affect viability of Miapaca-2 pancreatic cancer cells with mutant p53 (Figure 2G). In normal fibroblasts, WI38-hTERT cells, R2 also did not significantly affect viability (Figure 2H). Thus, the lead compound R2 significantly decreased the viability of cancer cells with wild type p53, without a significant decrease of viability in normal human fibroblasts and in cancer cells with mutant p53.

### The R2 compound bound the FAK N-terminal domain and disrupted the interaction of FAK and p53

**Figure 3:**
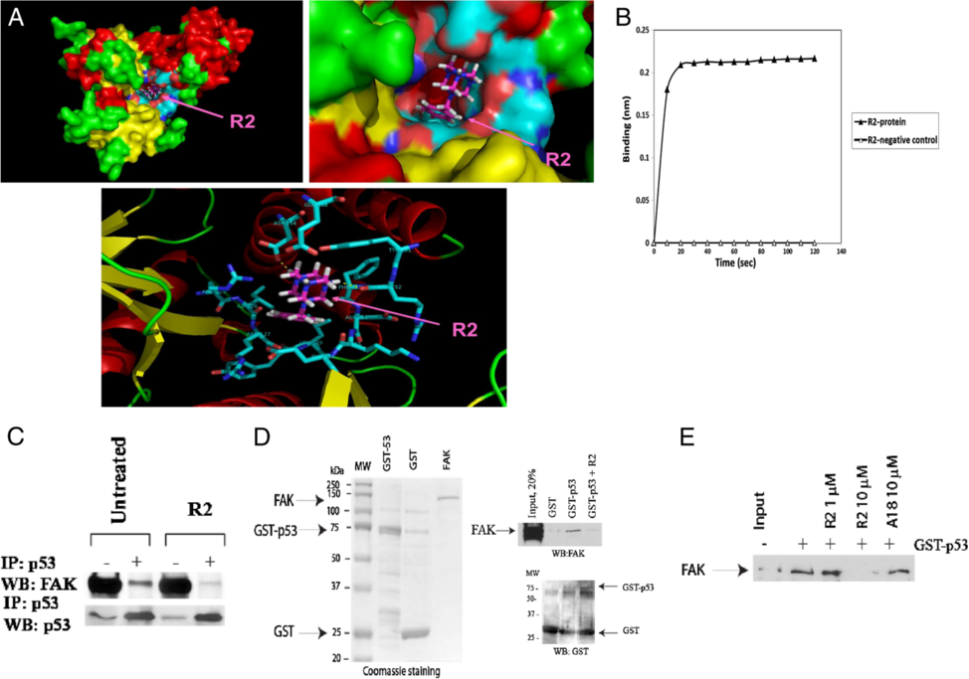
**R2 bound to the FAK-N-terminal domain and disrupted interaction of FAK and p53 proteins. A.** Upper panel. R2 compound docked into the FAK-NT protein. Lower panel: Zoomed image. The Blue color shows area of interaction. In the R2 compound, the blue color shows nitrogen and the red-oxygen and grey color shows carbon. The amino-acids of FAK-NT involved in interaction with R2 are shown in blue color. Hydrogen bonds are marked by yellow dashed color are between R2 compound and FAK amino-acids, Asp154 and Arg252. **B.** The R2 compound directly bound FAK-N-terminal domain by Octet Binding assay. Binding is observed with R2 and FAK-NT, but not with the negative control buffer. **C.** Immunoprecipitation showed that R2 disrupted binding of FAK and p53 proteins. The immunoprecipitatioon of p53 was performed after treatment of HCT116 cells with R2 at 100 μM for 24 h. Then Western blotting was performed with FAK antibody to detect complex of p53 with FAK. The binding was present in untreated cells, but not in R2-treated cells. Plus (+) marked immunoprecipitation; and minus (−) marked no immunoprecipitation. **D.** Pull-down assay demonstrated that R2 disrupted FAK and p53 complex. Left panel: Recombinant proteins andbaculoviral FAK (marked by arrows). Right panel: Pull-down assay with recombinant GST-p53 and FAK protein demonstrated binding of FAK and p53 proteins. The R2 compound disrupted the binding of FAK and p53 proteins. Upper panel: Western blotting with FAK antibody. Lower panel: Western blotting with GST antibody. **E.** R2 disrupted the binding of FAK and p53 proteins in a dose-dependent manner, while a negative control compound (A18), which was not targeting FAK-p53 interaction did not. The pull-down assay was performed as in Figure 3D with 1 and 10 μM of R2 and with 10 μM of A18 (negative control).

We performed computer modeling of the R2 compound docked into the FAK-NT region involved in interaction with p53 protein (Figure 3A). The R2 compound effectively docked into the FAK-NT domain (Figure 3A upper panels; zoomed image, lower panel).

To detect direct binding of R2 to the N-terminal domain of FAK, we isolated human N-terminal domain of FAK and performed Real-time binding assays with R2 compound using ForteBioOctet Red384 system (Figure 3B). The assay demonstrated that R2 directly bound to the FAK-NT protein, but not to the negative control (Materials and Methods) (Figure 3B). In addition, we performed by Octet assay kinetic analysis of association and dissociation of FAK and p53 proteins, either without R2 or with three different doses of R2 (Additional file 3: Table S1). The increased doses of R2 increased dissociation constant K_D_ of FAK and p53 protein interaction, supporting disruption of FAK and p53 complex by R2 in a dose-dependent manner.

To test disruption of FAK and p53 binding by R2 in cells, we performed immunoprecipitation (IP) of FAK and p53 proteins in HCT116 p53^+^/^+^ cells without R2 and with R2 (Figure 3C). While we detected the complex of FAK and p53 by IP in untreated cells (Figure 3C), we did not detect this complex in R2-treated cells. Thus, the R2 compound disrupted the interaction of FAK and p53 in HCT116 cells (Figure 3C). To test that R2 directly disrupted the binding of FAK and p53 proteins, we performed pull-down assays using purified recombinant baculoviral FAK, GST and GST-p53 proteins (Figure 3D, left panel). The pull-down assay clearly showed that FAK bound to p53 without R2, but there was no binding in the presence of R2 (Figure 3D, right panel). R2 disrupted the binding of FAK and p53 in a dose-dependent manner, while the negative control compound (A18),[32] which did not bind the FAK-p53 region did not disrupt the binding of FAK and p53 (Figure 3E). Thus, R2 bound FAK-NT and directly disrupted the binding of FAK and p53 proteins *in vitro* and *in vivo*.

### The R2 small molecule compound reactivated p53-transcriptional activity with p21, Mdm-2 and bax targets

**Figure 4:**
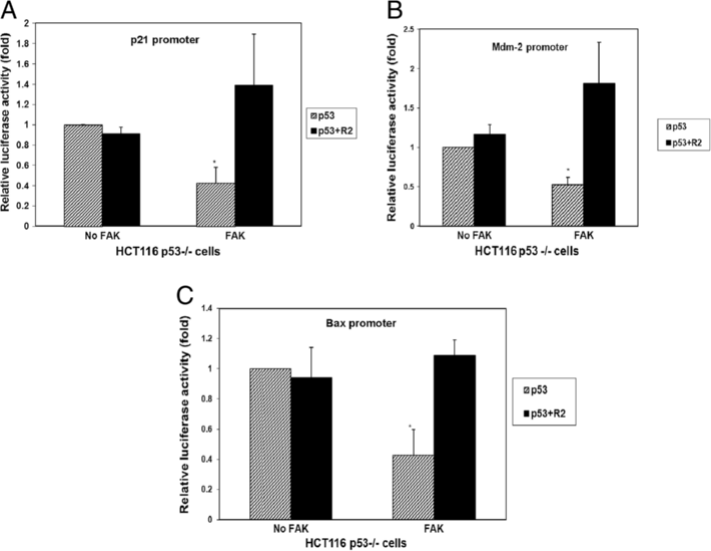
**R2 increased and reactivated p53 transcriptional activity that is inhibited by FAK. A.** Reactivation of p53 activity with p21 target by R2. The dual luciferase assay was performed in HCT116 p53−/− cells co-transfected with p53 and p21 promoter either without FAK plasmid without R2 or with 25 microM R2 treatment or with FAK plasmid without and with R2 treatment. The dual luciferase assay was performed as described in Materials and Methods. R2 compound reactivated p53 activity with p21 target inhibited by FAK. **B.** Reactivation of p53 activity with Mdm-2 target. The same assay as in Figure 4 A was performed with Mdm-2 promoter. R2 reactivated p53 activity with Mdm-2 target that was inhibited by FAK. **C.** Reactivation of p53 activity with Bax target. The same assay as in Figure 4A, B was performed with Bax promoter. R2 compound re-activated p53 activity with Bax target inhibited by FAK. *p<0.05, p53 activity with FAK versus no FAK, no R2 treatment, Student’s t-test.

To study the effect of R2 compound on p53-dependent signaling, we tested the effect of R2 on p53-regulated transcriptional targets, such as p21, Mdm-2, and Bax. We have shown before that overexpression of FAK plasmid blocked the transcriptional activity of p53 through interaction with p53 protein [18]. To test if disruption of the FAK and p53 interaction by R2 de-repressed p53 transcriptional activity, we co-transfected HCT116 p53^-^/^-^ cells with p53 plasmid and p21 promoter luciferase plasmid in the presence of R2 compound either without FAK plasmid or with the FAK plasmid. After 24 hours we added R2 at 25 μM and compared its effect with untreated cells. FAK blocked p53-induced p21 activity (Figure 4A), while treatment with R2 compound reversed this inhibition and re-activated p53-activity of the p21 target (Figure 4A). The same reactivation of p53 was demonstrated by R2 with Mdm-2 target (Figure 4B) and Bax target (Figure 4C). This effect was specific and not observed with the negative control compound M13, that targeted the FAK-MDM-2 interaction [33], but not the FAK and p53 interaction (Additional file 4: Figure S4). Thus, R2 specifically targeted FAK and p53 interaction and re-activated p53 targets: p21, Mdm-2, and Bax promoters.

### The R2 small molecule compound increased expression of p53-targets in a p53-dependent manner

**Figure 5:**
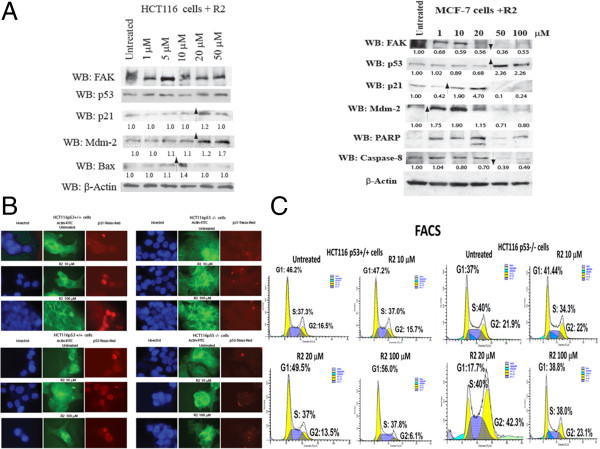
**R2 induced expression of p53 targets. A.** Induction of p53 targets in HCT116 and MCF-7 cells. The HCT116 p53^+^/^+^ cells (left panel) and MCF-7 (right panel) were treated with different doses of R2 and Western blotting was performed with p53, Mdm-2, Bax, PARP-1 and caspase-8 antibodies. R2 induced expression of p53 targets in a dose-dependent manner in HCT116 and MCF-7 cells. The affected proteins by R2 are shown by arrows. The densitometry quantitation was performed with Scion Image software. The protein level was measured and expressed relatively for the beta-actin control, and then normalized to untreated sample, which was equal to one. **B.** Immunostaining demonstrated that R2 activates p21 and increased nuclear localization of p53 and p21 proteins in HCT116 p53^**+**^/^**+**^ cells, but not in p53^-^/^-^ cells. Immunostaining with primary p21 (upper panel) or p53 (lower panel) and with secondary Texas-Red conjugated antibodies was performed on HCT116 p53^+^/^+^ and p53^-^/^-^ cells either untreated or treated with R2. The Phalloidin-FITC stained actin was used to observe cell morphology. R2 increased nuclear p53 and p21 in HCT116p53^+^/^+^ cells treated with R2 in contrast to HCTp53^-^/^-^ cells. **C.** R2 increased G1 arrest in R2-treated cells. Flow Cytometry analysis was performed as described in Materials and Methods on HCT116 p53^+^/^+^ and p53−/− cells that were either untreated or treated with different doses of R2 for 24 h. R2 increased G1-arrested cells and decreased G-2 arrested cells in p53^+^/^+^ cells but not p53^-^/^-^ cells.

To study the effect of R2 on p53 and p53-regulated targets, we performed Western blotting on HCT116 p53 treated cells with different doses of R2. We treated cells with different doses of R2 from 1 to 50 μM for 24 hours and performed expression analysis of p53 and its targets: p21, Mdm-2, and Bax (Figure 5A). R2 increased expression of p53 targets: p21, Mdm-2 and Bax in a dose-dependent manner in HCT116 cells (Figure 5A, left panel). In addition, we treated wild type p53 breast cancer MCF-7 cells with R2 (Figure 5A, right panel). R2 also increased p21 and Mdm-2 levels and at higher doses caused PARP-1 cleavage and caspase-8 activation in MCF-7 cells. In contrast to cancer cells with wild type p53, there was no up-regulation of Mdm-2 and p21 in SW620 colon cancer cells with mutant p53 (not shown). Thus, R2 increased the expression of p53 and its targets in a dose-dependent manner in cancer cells with wild type p53.

### The R2 small molecule compound increased nuclear localization of p21 and p53 and increased G1-arrest in HCT116 cells a p53-dependent manner

To detect the effect of R2 on p21 and p53 localization and activation, we performed immunostaining of p21 and p53 in HCT116p53^+^/^+^ and HCTp53^-^/^-^ cells that were either untreated or were treated with R2. We detected activation of p21 and increased nuclear localization by immunostaining of p21 in R2-treated HCT116p53^+^/^+^ (Figure 5B, upper panel). The activation and nuclear localization of p21 was observed in HCT116p53^+^/^+^ cells, but not in p53-negative cells, indicating p53-dependent activation of p21 by R2. Increased nuclear localization of p53 was observed in HCT116p53^+^/^+^ cells treated with R2, but was not detected in the negative control HCT116p53^-^/^-^ cells (Figure 5B, lower panel). Thus, R2 activated p53-targets in a p53-dependent manner.

We also performed cell cycle analysis of R2-treated and untreated HCT116 p53^-^/^-^ and p53^+^/^+^ cells by FACS (Figure 5C). We treated HCT116 cells with 10, 20, and 100 μM of R2 for 24 hours and then performed analysis of the cell cycle. We detected a significant dose-dependent increase of G1-arrest in R2-treated HCT116 p53^+^/^+^ cells from 46% in untreated cells to 56% at 100 μM of R2 (p<0.05). We also observed a decrease of G2-phase in these cells from 16% in untreated to 6% in R2-treated, but not in HCT116 p53^-^/^-^ cells (Figure 5C). Thus R2 activated the p53-target, p21, and increased G1 arrest in HCT116 cells in a p53-dependent manner.

### The R2 compound significantly decreased tumor growth, and up-regulated p21 expression in HCT116 tumor xenografts in a p53-dependent manner

**Figure 6:**
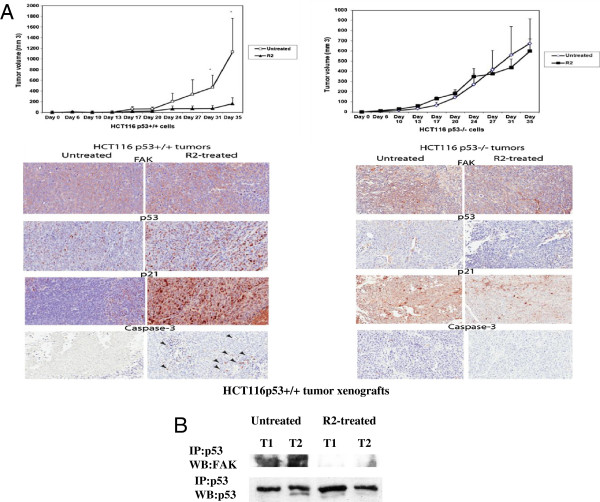
**R2 significantly decreased tumor growth and activated p21 in HCT116 p53**^**+**^**/**^**+ **^**but not in HCT116 53**^**-**^**/**^**- **^**tumor xenografts *in vivo. *A.** Upper panel: HCT116 p53^+^/^+^ and HCT116 p53^-^/^-^ cells were injected subcutaneously into the right and left leg flanks respectively. The control untreated mice were injected subcutaneously with 1xPBS. The treated group of mice was injected subcutaneously with 60 mg/kg of R2. In the case of R2-treated HCT116p53^+^/^+^ xenografts tumor volume decreased significantly (Student’s t-test, p<0.05, marked by asterisk), while HCT116p53^-^/^-^ xenograft tumor volume was not decreased in p53^-^/^-^ xenografts. Lower panel: R2 caused activation of p21 in HCT116p53^+^/^+^ tumors, but not in HCT116p53^-^/^-^ xenograft tumors. R2 increased p21 expression and activated caspase-3 in HCT116 p53^+^/^+^ xenografts but not in HCT116 p53^-^/^-^ xenografts. **B.** R2 disrupted FAK and p53 complex in HCT116p53^+^/^+^ xenografts. R2 disrupted FAK and p53 complex in HCT116 p53^+^/^+^ xenografts. We immunoprecipitated p53 in tumor xenograft samples and performed Western blotting with FAK antibody in untreated and R2-treated tumor xenografts. The complex of FAK and p53 was present in untreated xenografts, while the complex was not detected in R2-treated xenografts. Two representative tumors are shown for each group.

To test the effect of R2 on tumor growth *in vivo*, we subcutaneously injected isogenic HCT116 p53^+^/^+^ and HCTp53^-^/^-^ cells in the same mice into their right and left sides, respectively, and then treated them, with R2 and measured xenograft tumor growth (Figure 6A, upper panels). R2 significantly decreased tumor volume in HCT116 p53 ^+^/^+^ mice xenografts (Figure 6A, left upper panel), while it did not significantly decrease tumor growth in HCTp53^-^/^-^ xenografts (Figure 6A, right upper panel). We analyzed tumors from HCT116 p53^+^/^+^ xenografts and detected up-regulated expression of p21 in a p53-dependent manner: R2 increased p21 in HCT116 p53^+^/^+^ xenografts but not in p53^-^/^-^ xenografts, while it did not affect FAK and p53 protein levels (Figure 6A, lower panels). We also observed activation of caspase-3 in HCT116 p53^+^/^+^, but not in p53^-^/^-^ xenografts, consistent with a significant decrease of HCT116 p53^+^/^+^ xenograft tumor growth. Western blotting demonstrated increased p21 in HCT116 p53^+^/^+^ xenograft tumors but not in p53^-^/^-^ xenograft tumors (not shown). In addition, we performed immunoprecipitation of p53 and FAK in untreated and R2-treated HCT116p53^+^/^+^ xenografts and detected disruption of FAK and p53 complex in the HCT116 p53^+^/^+^ xenografts (Figure 6B). Thus, R2 blocked tumor growth, disrupted FAK and p53 and re-activated p53 by up-regulating p21 in HCT116 p53^+^/^+^ xenografts *in vivo*. Furthermore, the p53 specificity of R2 was confirmed with the lack of effect in the control p53 negative xenografts in each animal.

### The R2 sensitized cancer cells to doxorubicin and 5-fluorouracil

**Figure 7:**
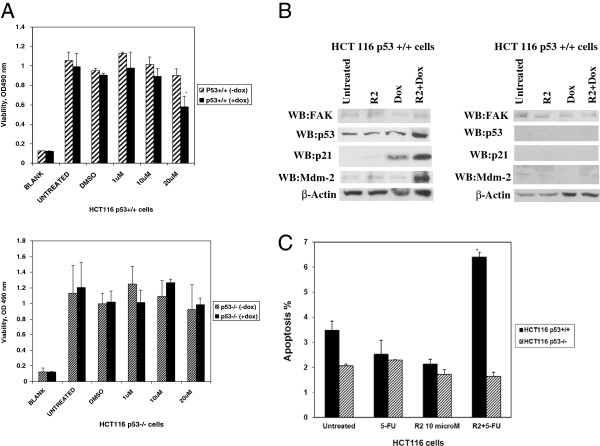
**R2 sensitized HCT116 cells to doxorubicin or 5 fluorouracil treatments. A.** MTT assay was performed on HCT116p53^+^/^+^ cells (upper panel) or HCT116p53^-^/^-^ (lower panel) either with doxorubicin alone (0.5 μg/ml), with different doses of R2 alone (−dox) or with combination of doxorubicin and R2 together (+dox). The combination of R2 and doxorubicin decreased colon cancer viability in a p53-dependent manner more effectively than each inhibitor alone in HCT116p53^+^/^+^ cells (upper panel), but not in HCT116 p53^-^/^-^ cells (left lower panel). * p<0.05, Student’s t-test R2 plus doxorubicin versus R2 without doxorubicin treatment. **B.** Western blotting on R2, Doxorubicin and R2 plus Doxorubicin-treated HCT116 cells. Cells were treated for 24 hours either with R2 (1 μM) or Doxorubicin (0.5 μg/ml) or a combination of R2 and Dox. Western blotting demonstrated that R2 increased p21, and Mdm-2 in HCT116p53^+^/^+^ cells and increased p21 was more effective in the case of R2+Dox treatment compared with each agent treatment alone. This effect was not observed in HCT116^-^/^-^ cells. **C.** Combination of 5-fluoroiracil (5-FU) and R2 increased apoptosis in HCT116 cells more significantly than each agent alone. Treatment of cells with R2 alone (10 μM), 5-FU alone (0.2 mM) or with both inhibitors together was performed on HCT116 p53^+^/^+^ and p53^-^/^-^ cells for 24 h. Apoptosis was analyzed by Flow Cytometry assay. R2 increases apoptosis in HCT116 p53^+^/^+^ cells treated with R2 in combination with 5-FU, but not in p53^-^/^-^ cells. Bars represent the average of apoptosis from two independent experiments ± standard deviations. *p<0.05, Student’s t-test.; R2+5-FU versus Untreated, R2-treated, and 5-FU-treated cells.

To test the effect of R2 on cancer cell viability in combination with chemotherapy, we treated HCT116 p53^+^/^+^ and HCT53^-^/^-^ cells with R2 alone, doxorubicin alone, or with R2 and doxorubicin together (Figure 7A). R2 sensitized HCT116 p53^+^/^+^ cells to doxorubicin (Figure 7A, upper panel) but not HCT116 p53^-^/^-^ cells (Figure 7A, lower panel). Western blotting detected increased p53, p21 and Mdm-2 expression in the case of a combination of R2 and doxorubicin compared with each agent alone in HCT116p53^+^/^+^ cells (Figure 7B, left), but not in HCT116p53^-^/^-^ cells (Figure 7B, right). Thus, R2 sensitized colon cancer cells to doxorubicin in a p53-dependent manner. The same sensitizing effect as in the case of R2 and doxorubicin was observed in the combination of R2 and 5-fluorouracil, where FACS analysis demonstrated significantly increased apoptosis in the case of the combination of R2 and 5-fluorouracil in HCT116p53^+^/^+^ cells, but not in p53^-^/^-^ cells (Figure 7C). Thus, R2 sensitized cancer cells to different chemotherapy drugs, which can be important for developing FAK-p53 combination therapy approaches.

## Discussion

In this report, we have demonstrated that the binding between FAK and p53 can be disrupted within a small molecule mimetic that targeted their interaction site. This released normal p53 and activated its downstream targets, including MDM-2, p21, and Bax. Furthermore, these effects were highly specific for p53 as demonstrated in the isogenic HCT-116 colon cancer cell lines that differed only in the presence or absence of p53.

Our results are consistent with the role of FAK in binding pro-apoptotic proteins in cancer cells to inactivate their normal function and thus provide a growth advantage to the tumor cell. This model for one of FAK’s functions has been termed sequestration by Frisch [15,41,42]. In addition to p53, FAK binds other proapoptotic proteins such as RIP [43] and NF1 [44]. Given the massive overexpression of FAK in tumor cells [7], binding and sequestering these tumor suppressive proteins appears to be an important part of FAK’s function in survival signaling. We have shown that FAK inhibits p53 transcriptional activity [18] and disruption of FAK and p53 de-repressed activity of p53 to activate its downstream targets.

The binding of FAK and p53 is one axis in a tripartite complex between FAK, p53 and MDM-2 [19]. The p53-MDM-2 interaction has been extensively studied and small molecules have been created that disrupt their binding [45]. They have been tested in both preclinical as well as early-stage clinical trials. Our group has recently reported the development of small molecules that disrupt the FAK-MDM-2 interaction [33]. The combination therapy approach can be studied in the future with FAK-p53-Mdm-2 inhibitors.

We have described R2 as a lead compound that provides “proof of principle” that the FAK and p53 interaction can be disrupted by small molecules with reactivation of p53 activity and resultant cytotoxicity to HCT116 cells. In addition, the disruption of FAK-p53 binding and reactivation of p53 activity was seen in the tumor samples themselves, demonstrating the specificity of R2 targeting. The reactivation of p53 in HCTp53^+^/^+^ tumors also had a sensitizing effect to chemotherapy that will be important for future therapeutic efforts. In fact, we were able to show that a combination of doxorubicin or 5-fluorouracil and R2 was more effective in decreasing colon cancer viability than either one alone. This may be the result of R2 making the cancer cells more sensitive to cytotoxic therapy, or it may be the effects of chemotherapeutics like doxorubicin that have been shown to induce expression of p53 [46].

These results also demonstrate the importance of the non-kinase or scaffolding function of FAK. There is a mounting body of evidence that the non-kinase functions of FAK are separate, but as significant as its kinase function [47,48]. For example, FAK−/− knock-out mice had shorter survival than kinase-dead mice [49,50], additionally supporting the concept that FAK has important functions in addition to its kinase-dependent function. In fact, recent reports demonstrated that this scaffolding function of FAK is very important for cancer cell functions [48]. Thus, targeting the kinase-independent function of FAK such as the interaction between FAK and p53 is a novel approach that is complementary to existing therapeutic strategies that target the FAK kinase function.

## Conclusions

In conclusion, we isolated the novel small molecule compound Roslin 2 and demonstrated that it disrupted the FAK and p53 interaction and reactivated p53 transcriptional activity with its downstream targets. Disruption of FAK and p53 and reactivation of p53 with R2 compound decreased cancer cell viability and clonogenicity and inhibited tumor growth *in vivo* in a p53-dependent manner. In addition, R2 compound sensitized cancer cells to chemotherapy. These data define a novel approach to reactivating p53 by disrupting the complex of FAK and p53 with the small molecule compound R2 that can be effectively used for future pre-clinical and clinical therapeutic models.

## Abbreviations

FAK: Focal Adhesion Kinase; Mdm-2: mouse double minute homolog; R2: Roslin 2 compound.

## Competing interests

Dr. Golubovskaya and Dr. Cance are Co-Founders and shareholders of *CureFAKtor Pharmaceuticals*. All other authors declared no conflict of interest.

## Authors’ contributions

VG and WC contributed to the conception, design, analysis, and interpretation of data and were involved in writing of the manuscript. BH and MZ performed *in vitro* viability, clonogenicity, cell cycle, biochemical assays and *in vivo* mice experiments. AM and DO performed computer modeling and docking experiments. CM developed and performed immunohistochemical staining analysis of xenograft samples. All authors read and approved the final manuscript.

## Authors’ information

WC is a Chair of Surgical Oncology Department and Surgeon-in-Chief, Professor, Leader of Experimental Therapeutics Group, Roswell Park Cancer Institute, NY. VG is an Associate Professor of Surgical Oncology and Member of the Experimental Therapeutics Group of Roswell Park Cancer Institute, NY. WC and VG are Active Members of AACR.

## Pre-publication history

The pre-publication history for this paper can be accessed here:

http://www.biomedcentral.com/1471-2407/13/342/prepub

## Supplementary Material

Additional file 1: Figure S1 The screening of R compounds in different cell lines. A. The viability MTT assay with R compounds was performed in A375 melanoma cells with wild type p53. B. Viability MTT assay with small molecules targeting FAK-p53 interaction in FAK^-^/^-^p53^-^/^-^ MEF cells. To test specificity for FAK and p53 interaction MTT assay with R compounds was performed in normal FAK^-^/^-^p53^-^/^-^ MEF cells. Most of compounds did not affect the viability of the FAK−/−p53−/− MEF cells except for R9, R10, R12, and R13 compounds. C. The MTT assay with R compounds on Panc-1 pancreatic cancer cell line with mutant p53. Most compounds did not significantly affect viability of PANC-1 cells, except of R13 compound. Click here for file

Additional file 2: Figure S2 R2 is the most effective compound to decrease clonogenicity. The clonogenicity assay was performed with the R2, R5 and R7 compounds (structures are shown on left panels) and identified that R2 is the most effective in decreasing cancer clonogenicity (right panels). Click here for file

Additional file 3: Table S1 The dose-dependent effect of R2 on kinetics of FAK and p53 protein interaction by Octet assay. Click here for file

Additional file 4: Figure S4 No induction of p53 activity with control compound M13, which did not target FAK-p53 interaction. The control small molecule compound, M13 did not induce p53 activity of p21 target in contrast to R2 compound. Click here for file
