# Supplementary figures and images for: Disruption of focal adhesion kinase and p53 interaction with small molecule compound R2 reactivated p53 and blocked tumor growth
Source: BMC Cancer. 2013 Jul 11;13:342. doi: 10.1186/1471-2407-13-342 (PMC3712010; doi:10.1186/1471-2407-13-342)

## Slide 1
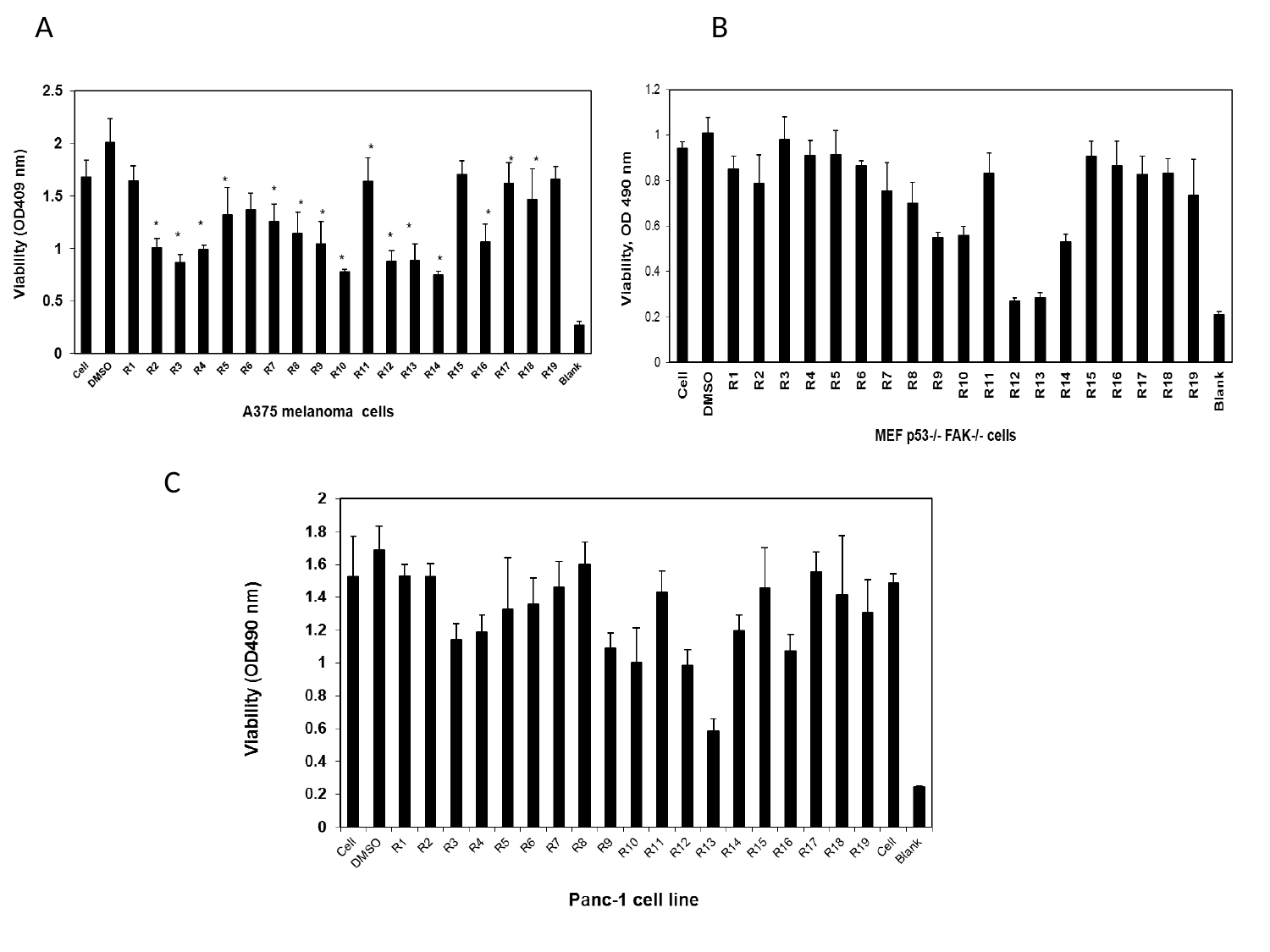

A
B
C

Supplement: Additional file 1: Figure S1 — The screening of R compounds in different cell lines. A. The viability MTT assay with R compounds was performed in A375 melanoma cells with wild type p53. B. Viability MTT assay with small molecules targeting FAK-p53 interaction in FAK-/-p53-/- MEF cells. To test specificity for FAK and p53 interaction MTT assay with R compounds was performed in normal FAK-/-p53-/- MEF cells. Most of compounds did not affect the viability of the FAK−/−p53−/− MEF cells except for R9, R10, R12, and R13 compounds. C. The MTT assay with R compounds on Panc-1 pancreatic cancer cell line with mutant p53. Most compounds did not significantly affect viability of PANC-1 cells, except of R13 compound. [file 1471-2407-13-342-S1.pptx]

## Slide 1
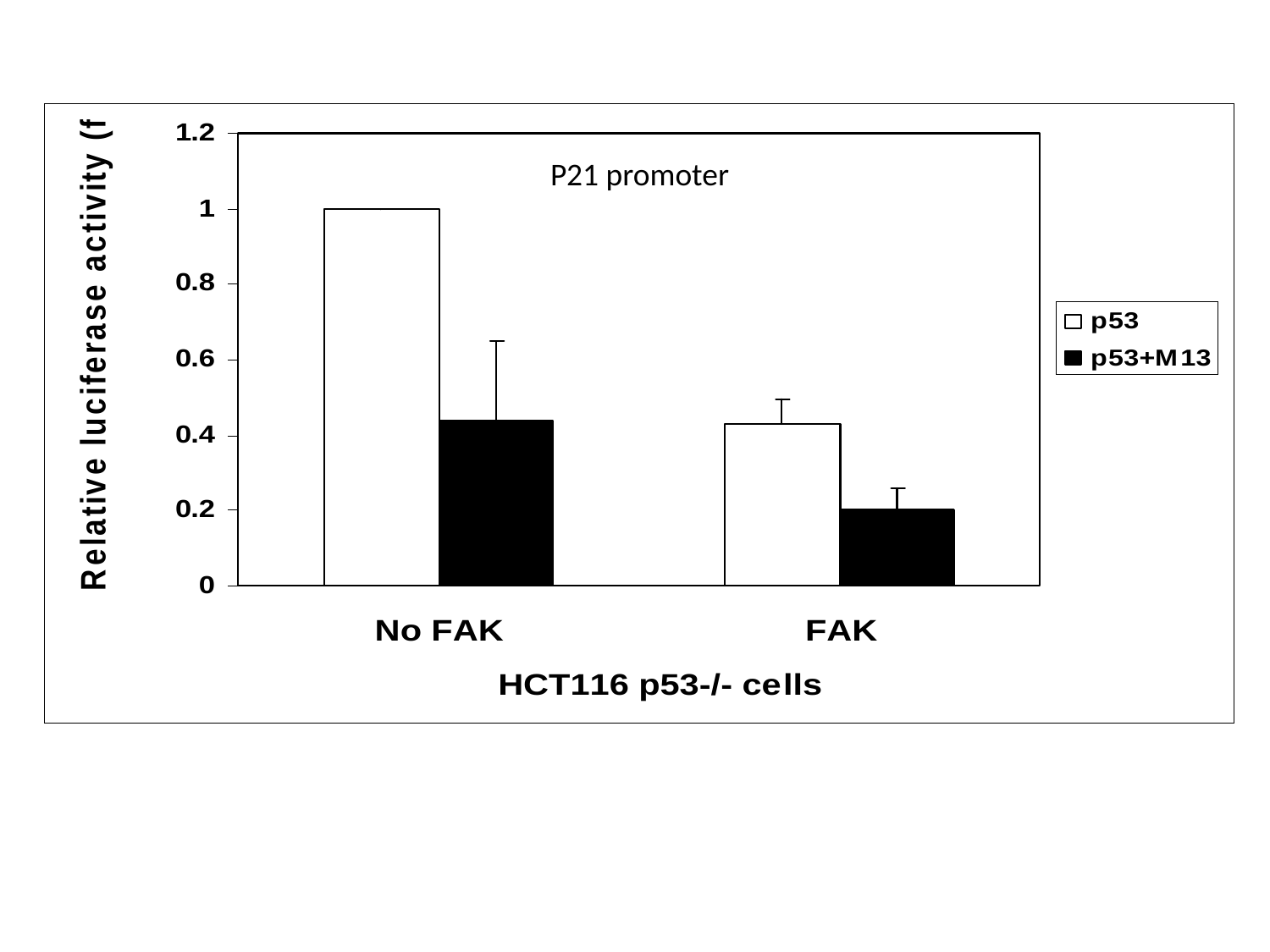

P21 promoter

Supplement: Additional file 4: Figure S4 — No induction of p53 activity with control compound M13, which did not target FAK-p53 interaction. The control small molecule compound, M13 did not induce p53 activity of p21 target in contrast to R2 compound. [file 1471-2407-13-342-S4.pptx]
