# Supplementary material for: Disruption of focal adhesion kinase and p53 interaction with small molecule compound R2 reactivated p53 and blocked tumor growth
Source: BMC Cancer. 2013 Jul 11;13:342. doi: 10.1186/1471-2407-13-342 (PMC3712010; doi:10.1186/1471-2407-13-342)
Supplement: Additional file 2: Figure S2 — R2 is the most effective compound to decrease clonogenicity. The clonogenicity assay was performed with the R2, R5 and R7 compounds (structures are shown on left panels) and identified that R2 is the most effective in decreasing cancer clonogenicity (right panels). [file 1471-2407-13-342-S2.pptx]

## Slide 1
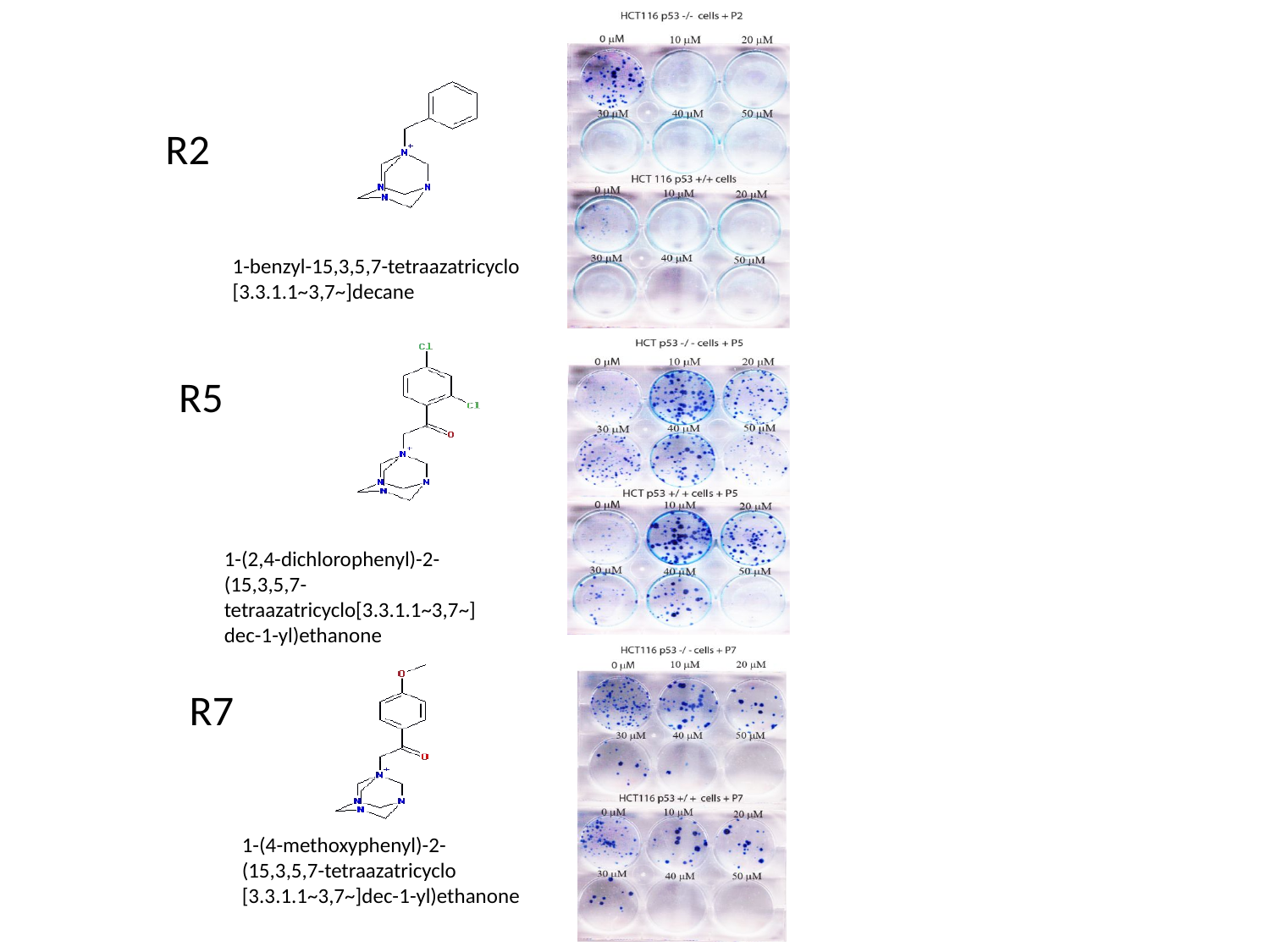

R2
1-benzyl-15,3,5,7-tetraazatricyclo
[3.3.1.1~3,7~]decane
R5
R7
1-(4-methoxyphenyl)-2-
(15,3,5,7-tetraazatricyclo
[3.3.1.1~3,7~]dec-1-yl)ethanone
1-(2,4-dichlorophenyl)-2-
(15,3,5,7-tetraazatricyclo[3.3.1.1~3,7~]
dec-1-yl)ethanone
