# Supplementary material for: Disruption of focal adhesion kinase and p53 interaction with small molecule compound R2 reactivated p53 and blocked tumor growth
Source: BMC Cancer. 2013 Jul 11;13:342. doi: 10.1186/1471-2407-13-342 (PMC3712010; doi:10.1186/1471-2407-13-342)
Supplement: Additional file 3: Table S1 — The dose-dependent effect of R2 on kinetics of FAK and p53 protein interaction by Octet assay. [file 1471-2407-13-342-S3.docx]

**Table S1. The dose-dependent effect of R2 on kinetics of FAK and p53 protein interaction by Octet assay.**

| **Compound** | **k_dis_**  **(1/s)** | **k_on_**  (1/Ms) | **K_D_**  **(M)** |
| --- | --- | --- | --- |
| **No R2** | **4.76x10^-3^** | **8.76x10^4^** | **5.44x10^-8^** |
| **R2 111 μM** | **6.86x10^-3^** | **3.04x10^4^** | **2.27x10^-7^** |
| **R2 333 μM** | **2.35x10^-2^** | **5.00x10^4^** | **4.70x10^-7^** |
| **R2 1000 μM** | **8.40x10^-1^** | **9.95x10^4^** | **8.44x10^-6^** |

**Rate constants: K_dis_, k_on_ and K_D_** **were obtained from the kinetics analysis curves of association-dissociation of FAK and p53 complex either in the absence of R2 or with three different doses of R2 by Octet software, as described in Materials and Methods. R2 increased dissociation constant K_D_ in a dose-dependent manner.**
